# Ecohydrodynamics of Cold-Water Coral Reefs: A Case Study of the Mingulay Reef Complex (Western Scotland)

**DOI:** 10.1371/journal.pone.0098218

**Published:** 2014-05-29

**Authors:** Juan Moreno Navas, Peter L. Miller, Lea-Anne Henry, Sebastian J. Hennige, J. Murray Roberts

**Affiliations:** 1 Centre for Marine Biodiversity and Biotechnology, Heriot-Watt University, Edinburgh, United Kingdom; 2 Remote Sensing Group, Plymouth Marine Laboratory, Plymouth, United Kingdom; 3 Center for Marine Science, University of North Carolina, Wilmington, North Carolina, United States of America; 4 Scottish Association for Marine Science, Scottish Marine Institute, Oban, United Kingdom; University of Waikato (National Institute of Water and Atmospheric Research), New Zealand

## Abstract

Ecohydrodynamics investigates the hydrodynamic constraints on ecosystems across different temporal and spatial scales. Ecohydrodynamics play a pivotal role in the structure and functioning of marine ecosystems, however the lack of integrated complex flow models for deep-water ecosystems beyond the coastal zone prevents further synthesis in these settings. We present a hydrodynamic model for one of Earth's most biologically diverse deep-water ecosystems, cold-water coral reefs. The Mingulay Reef Complex (western Scotland) is an inshore seascape of cold-water coral reefs formed by the scleractinian coral *Lophelia pertusa*. We applied single-image edge detection and composite front maps using satellite remote sensing, to detect oceanographic fronts and peaks of chlorophyll *a* values that likely affect food supply to corals and other suspension-feeding fauna. We also present a high resolution 3D ocean model to incorporate salient aspects of the regional and local oceanography. Model validation using *in situ* current speed, direction and sea elevation data confirmed the model's realistic representation of spatial and temporal aspects of circulation at the reef complex including a tidally driven current regime, eddies, and downwelling phenomena. This novel combination of 3D hydrodynamic modelling and remote sensing in deep-water ecosystems improves our understanding of the temporal and spatial scales of ecological processes occurring in marine systems. The modelled information has been integrated into a 3D GIS, providing a user interface for visualization and interrogation of results that allows wider ecological application of the model and that can provide valuable input for marine biodiversity and conservation applications.

## Introduction

In a time of unprecedented global climatic change and human pressures in the ocean, there is an urgent need to understand marine ecosystems, their dynamics and the mechanisms that underpin their structure and functioning. Hydrodynamics are an important but inadequately considered constraint on marine ecosystems, despite much of the fauna and flora depending on adequate water flow for nutrient and food provision, oxygen supply and vital biological processes such as larval dispersal. Turbulent mixing, transport by waves and small scale circulation cells tend to increase ecosystem efficiency by augmenting particle encounter rates and giving species a better chance of finding a favorable environment [1]. Bottom layer turbulence and resuspension of eroded sediments help disperse food for benthic and demersal communities. Hydrodynamic processes operating on ecological timescales also affect biogeochemical interactions that in turn constrain chemical and biological properties of ecosystems [2],[3].

Ecohydrodynamic approaches investigate the hydrodynamics constraints on ecosystems across different temporal and spatial scales [2] encompassing the physical, chemical and biological characteristics of the ocean layers that sustain marine ecosystems. Hydrodynamics around coral colonies and entire coral reefs span a range of spatial scales in which fluid motions operate, starting with large-scale flow phenomena such as eddies produced by reef wakes, to smaller scale turbulent features created by reef topology and to finer spatial scales of flow occurring around single coral colonies and polyps [4], [5]. Lateral and vertical advection of particles also plays a significant role in the functioning of coral ecosystems, and reef geometry plays a key part in determining patterns and rates of coral reef fish larval transport, reef connectivity and transport of other particles [6],[7].

Cold-water reef framework-forming scleractinian corals such as the globally distributed *Lophelia pertusa* (Linnaeus 1758) lack symbiotic algae, feeding solely on particles advected by water motion [8],[9]. Hydrodynamics thus play an important role in the transport of food to some of the most biologically diverse ecosystems on the deep seafloor, and are themselves mediated by processes including internal waves and tidal currents, all of which can locally enhance food supply. For example, in continental margin settings, cold-water coral reefs occur where geostrophic currents and internal waves steepen and break along the continental slope [10]. Seasonal changes in current, temperature and salinity patterns also occur in areas due to seasonal changes in the slope current [11],[12]. Near-seabed currents are significantly influenced by local topography and the patchy growth of the corals is often related to the varied speed and direction of local currents [11]. Active sediment transport by strong local near-bed currents helps prevent coral burial and also supplies sediment that becomes baffled in the dead coral framework contributing to the vertical growth of mounds [11],[13],[14].

Recent studies in these ecosystems suggest a coupling between the reef fauna and the surface productivity due to the local hydrography and sedimentary dynamics [10],[15],[16]. *Lophelia pertusa* is frequently associated with areas where surface waters show above average primary productivity [17]. In some continental shelf areas, domes of dense nutrient-rich waters can be generated over the banks, enhancing surface productivity that can be delivered down to the corals via several oceanographic dynamics [14]. Surface production and copepod species appear to be important food sources due to the fatty acid compositions of these animals and phytodetritus from the surface [11],[18]. The annual cycle in primary flux as well as the seasonal variability in near-bed hydrodynamic might also control coral growth and reproduction [19]. The ecohydrodynamic conditions around cold-water coral reefs therefore determine both the supply of food particles and recruits to the reef.

Remote sensing provides frequent views of a wide range of physical and biological processes occurring at the sea surface that may influence the ecology of corals. These processes include fronts, mesoscale eddies, currents, upwelling, phytoplankton and algal blooms. Three-dimensional (3D) hydrodynamic modelling can provide spatially explicit information on the key variables governing the oceanographic dynamics between surface phenomena and deep marine ecosystems with enough temporal resolution to give valuable information about turbulence, current regime and large scale oceanographic processes.

**Figure 1:**
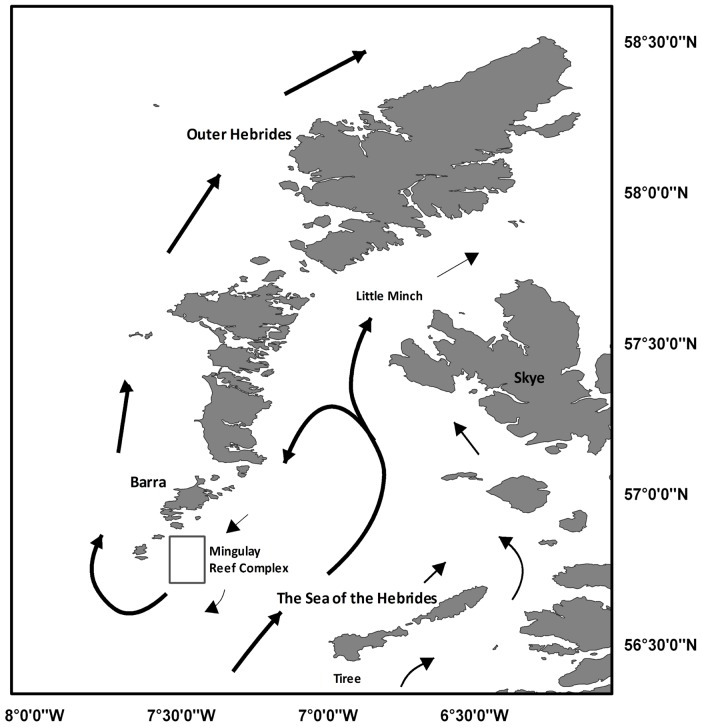
General current circulation. Surface circulation of the Hebridean island chain (adapted from ([29]).

Our study is the first to apply these principles to ecosystems engineered by reef framework-forming cold-water corals. Our main goal is to characterize the oceanographic conditions using remote sensing techniques and the development of a 3D hydrodynamic model in order to assess the ecological constrains in a cold coral reef at medium temporal and spatial scales. The Mingulay Reef Complex, located 13 km to the east of the island of Mingulay in the Sea of the Hebrides (western Scotland, Figure 1), was chosen as a case study since the area has been intensively studied since its discovery in 2003 [20],[21].

### Regional hydrodynamic setting

The Scottish western continental shelf has three water sources: Atlantic water propagating northward from the west of Ireland, Irish Sea water passing northward through the North Channel, and coastal water created by the high volume of river runoff from the Scottish mainland [22]–[27]. The overall circulation pattern comprises a net northward transport along the Scottish west coast, giving a northward flux of about 11×10^4^ m^3^ s^−1^ passing through the Little Minch [26] with variability superimposed by meteorological events [28]. Drifting buoys revealed a bifurcation of the northward coastal current in the Sea of the Hebrides: a water mass passes through the Little Minch and a recirculating southward current towards Barra Head [29] (Figure 1). The recirculated current includes two barotropic processes, tidal rectification and topographic steering of large scale flows, and a third mechanism whereby a baroclinic pressure gradient is associated with the observed dome of dense saline Atlantic water which extends into the centre of the channel from the south [30].

The regional oceanography is characterized by an intrusion of high salinity, high nutrient Atlantic water into the Sea of the Hebrides, and a weaker intrusion in the North Minch that produces a cyclonic circulation system [27],[29]. During summer months surface heating over topographic depression regions with weak tidal stirring, can lead to the formation of domes of cold dense bottom water trapped beneath the thermocline. The dense water dome is isolated horizontally from adjacent water by bottom fronts, which drive a cyclonic, near-surface circulation around the dome [29].

The seasonal water column characteristics and the cycles of physical and chemical properties of the whole Hebridean island chain are broadly repeated each year [27]. Sea surface temperature varies between 7–8 °C in spring with the warmest temperatures in autumn (13–14 °C). Deeper water temperatures show an intrusion of Atlantic Water into the southwest corner of the Sea of the Hebrides [22]–[27]. Salinity and nutrient levels followed a positive east to west gradient, with higher values in the west mainly caused by freshwater discharge from the main Scottish landmass. Elevated chlorophyll-*a* levels in surface coastal waters were found during spring, suggesting a phytoplankton bloom that begins near the Scottish mainland, Little Minch and the Sea of the Hebrides [27],[30],[31].

The distribution of physical and chemical parameters in the Sea of the Hebrides reveal a weak thermal stratification in spring and a strong stratification during summer and autumn, the column water being well mixed during winter [27]. The salinity was always >35 ppt throughout the year, reaching the maximum of 35.3 ppt during spring, summer and autumn. The density distribution confirms a pool of dense Atlantic water throughout spring and autumn, with surface nutrient depletion of nitrate and silicate during summer and autumn. High nitrate phosphate and silicate concentrations correlated positively with high values of salinity except ammonia and chlorophyll-*a*, which were inversely correlated [27].

### Geological history and present-day biodiversity

The bedrock of the Mingulay reef complex comprises Mesozoic sediments and a Paleocene intrusion that have been eroded by the recent glaciation [32]. This created a seascape that contains hollows >250 m deep and rocky ridges that rise more than 100 m above the surrounding seafloor. The interaction between water currents and irregular topography has exposed bedrock and boulders in elevated positions with bare rock predominating on topographic highs. The sediments in the area are dominated by muddy sand with broken shells and variable gravel content, with patches of coarse sediments and gravel in the north and west of the Mingulay Reef Complex [20],[21].

Fifteen distinct habitats including *L. pertusa* reef and coral rubble were identified from the first acoustic backscatter, video and grab surveys of the Mingulay reefs [20] with further mapping in 2006 allowing a total area of coral reef and rubble habitat in the complex to be estimated at 5.4 km^2^ [33]. Over 500 macroinvertebrate species inhabit the reef complex, which form benthic communities that change across space due to interactions between changes in seabed terrain and current speed [34],[35]. Ecohydrodynamic phenomena are thought to partially explain why oviparous egg-laying sharks are using specific areas on the reef complex for spawning [36].

The area has been bathymetrically mapped using multibeam echosounders, revealing several reefs formed by the cold-water coral *Lophelia pertusa* [20],[21],[37]. The Mingulay reefs have developed intermittently during the Holocene with the oldest coral material so far dated from 7.7 ka [38]. A number of cases of apparently recent established coral colonies have been noted suggesting recruitment is still occurring [24]. Previous studies have shown that the Mingulay reefs have two dominant food supply mechanisms; a regular rapid downwelling of surface water delivering pulses of warm water and a periodic advection of high turbidity bottom waters [16]. In the Mingulay tidal regime, the hydraulic control of the flow over a bank can lead to a depression of the density structure downstream of it (hydraulic jump). When the tidal flow over the reef weakens and reverses, this depression propagates as an internal wave over the summit in the previously upstream direction, driving superficial waters downward and over the reef [16]. This sequence occurs on both tidal phases but is strongest on the flood to ebb because of the asymmetric profile of the tidal current. The higher turbidity in the advected water is most likely due to the fine grain sediment surrounding the reef area that becomes entrained in the bottom water because high frictional turbulence and accelerated tidal currents on the reef top enhance the delivery of particles suspended in deeper water [16].

### The three-dimensional (3D) water modelling system, MOHID

Three dimensional hydrodynamic modelling has been used extensively to understand the flow dynamics in both shallow and oceanic waters. There have been many detailed studies of the hydrodynamics of tropical coral reefs [4],[5] but only two previous studies used hydrodynamic models [10],[15] to examine the ecology of cold-water coral reefs.

The hydrodynamic model MOHID (Modelo Hidrodinámico) used in this study was developed by the Marine and Environmental Technology Research Centre (MARETEC) at Instituto Superior Técnico, Technical University of Lisbon. This model has been previously used in ocean circulation, transport and mixing simulations along the continental shelf edge, in oil spill management, operational oceanography, nutrient load and residence time models in several places around the world (see http://www.mohid.com/wiki/index.php?title=Mohid_Bibliography#2013).

The model solves the equations of a three-dimensional flow for incompressible fluids and an equation of state relating density to salinity and temperature [39]–[41].

The Cartesian coordinate framework [41] equations are as follows:
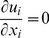
(1)

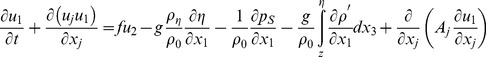
(2)

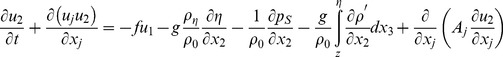
(3)

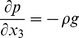
(4)Where (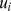) is the velocity component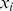, 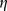 is the free surface elevation, 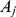 is the turbulent viscosity coefficient, 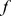 is the Coriolis parameter, 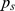 is the atmospheric pressure, 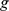 is the gravitational acceleration, 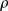 is the density and 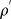 represents the density anomaly as the depth mean density minus the density at particular height. The density is calculated as a function of salinity and temperature using the equation of state [42].

The residual current 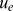 was estimated by averaging the horizontal flow 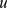 over the modelled time period 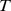:
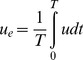
(5)


The nested system used in this model consists of two sub-components: a coarse-resolution outer model covering part of the Sea of the Hebrides with a horizontal resolution of roughly 300 m, and a fine-resolution inner model covering the Mingulay Reef Complex with a horizontal resolution of roughly 100 m. In order to ensure model stability and a smooth transition between modelled areas certain constrains are imposed following the suggested protocol for nesting a hydrodynamics model [43].

**Figure 2:**
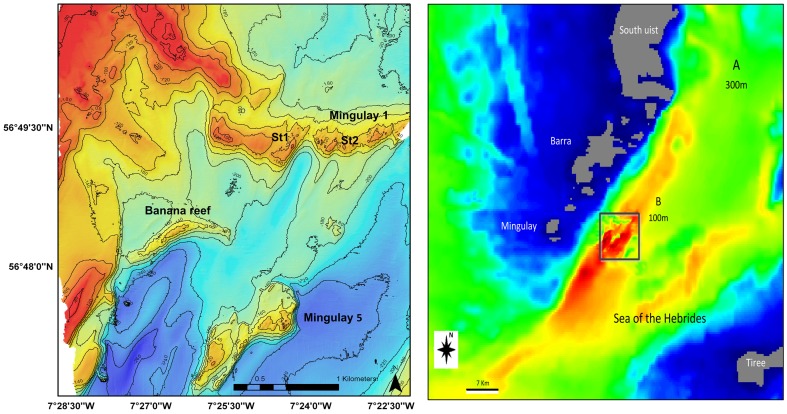
Detail maps of the study area. Bathymetry of Mingulay reef complex (left), and the bathymetry grid resolution used in the hydrodynamic model (right).

A bathymetry model was obtained from GEBCO and from two multibeam echosounder surveys carried out across the reef complex. The first survey led to the discovery of the Mingulay Reef Complex in 2003 and was carried out using a Kongsberg EM2000 multibeam echosounder [20]. The second survey in 2006 extended this area using a Kongsberg EM300 echosounder leading to the discovery of the Banana Reef [21],[37],[44]. These data were used to produce an initial GIS vector data layer followed by interpolation to 300 m and 100 m grid resolutions (Figure 2). An Arakawa C grid was used for spatial discretization [45] and in this study a sigma coordinate was chosen with eight vertical layers.

The modelled hydrographic parameters were derived for two sampling stations within the reef complex and were recorded over model runs of 15 days (6/07/06 to 21/07/06) covering a lunar tidal cycle and coinciding with the period during which measurements were available [16]. Two hydrologic time series over two days were selected (11-13/07/06 at Station 1 and 14-15/07/06 at Station 2); two data cross-sections of one day were separated for use in the calibration and validation processes, and independently for cross-validation. Vertical eddy viscosity/diffusivity was determined with a turbulence closure model selected from those available in the General Ocean Turbulence Model (GOTM) [46].

In this application a 3D model forced with tide was implemented. Water level imposed at the boundary was taken from the FES2004 global tide solution [47], which gave heights of tidal constituents as it was not possible to use local tide gauge information. The model incorporates the islands of South Uist, Barra, Mingulay and Tiree (see Figure 2) covering an area 100 km north-south and 80 km east-west.

We compared the tidal water elevation calculated from the tidal constituents as per FES2004 with modelled values of tidal elevations from MOHID. The parameters used in the model calculations are summarized in Table 1.

**Table 1:** Parameters used in the model calculations.

Physical parameter	Numerical value
Time step:	10 s
Grid mesh:	300,100 m
Horizontal cells (x,y):	(193,244) (114,126)
Vertical coordinate:	Sigma
Vertical layers:	8
Horizontal Eddy Viscosity:	4.3810 m^2^·s^−1^
Vertical Eddy Viscosity:	Derived from GOTM
Drag coeffcient:	0.0025
River discharges;	No
Salinity:	35 psu
Temperature:	11°C
Forced:	Tide from FES2004

Rates of residual flow were computed because this helps to understand long-term water exchange in the area. The residual current was estimated by averaging the horizontal flow over a modelled time period of 15 days (lunar tidal cycle).

For the validation process several descriptive statistics (mean, variance and standard deviation), the relative mean absolute error (RMAE) [48] and the Index of Agreement (IoAd) [49],[50] were calculated.

### Ocean colour

Local oceanic fronts were studied using both thermal and colour data from satellite observations. For thermal fronts, 10 years of Advanced Very High Resolution Radiometer (AVHRR) sea-surface temperature data from December 1998 to November 2008 were processed, comprising several passes per day over the UK at a maximum 1.1 km resolution. Chlorophyll-*a* fronts were processed using data from the Moderate Resolution Imaging Spectroradiometer (MODIS) sensor on the NASA Aqua satellite, from 2009 to 2011 at 1 km resolution. The composite front map technique was used to combine the location, gradient, persistence and proximity of all fronts observed over each month into a single map [51]. The monthly front maps were then aggregated into seasonal front climatologies to identify frequently occurring features [52],[53].

## Results

**Figure 3:**
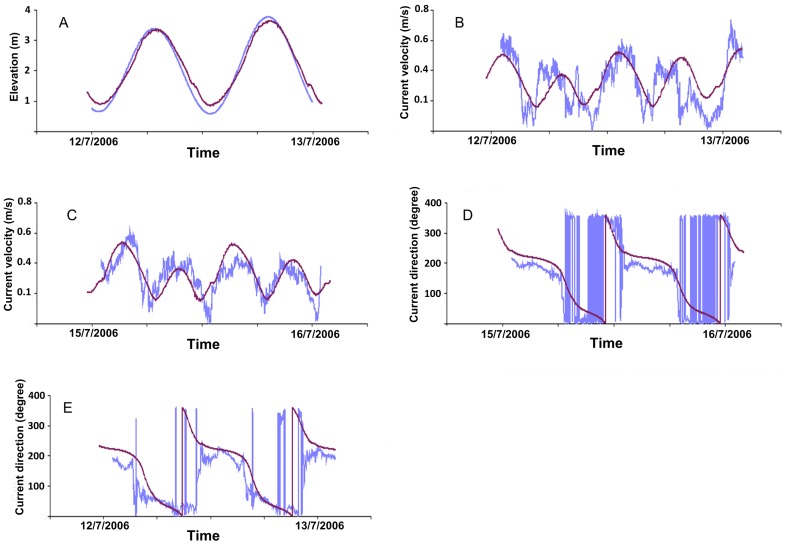
Model validation. The modelled hydrographic parameters were derived for two sampling stations within the reef complex and were recorded for model runs 15 days in duration (6/07/06 to 21/07/06). Time series of the intensity and current direction (blue line) and modelled (dark line) of one day at two stations St1 (B,E) and St2 (C,D) at the bottom and elevation measurements (A).

Figure 3 shows a time series of the water current intensity of one day used for validation. The direction and elevation measured (blue line) and modelled (dark line) for two stations St1 and St2 at the bottom using the lowest sigma layer with around 3 m of thickness. For sea surface elevation, the calculated values of IoAd were close to 1, and low values of RMAE revealed a good agreement between the prediction of the model [48] and the observations (table 2). A nested model with high resolution (mod100) was applied in detail at the Mingulay Reef Complex and produced similar current characteristic as given by the coarse resolution model (mod300). The water current intensity IoAd values varied from 0.64 and 0.84, suggesting that our model produced acceptable results. The values of mean, variance and standard deviation of the observed and modelled current intensity at the two stations were similar. The RMAE values were very low, suggesting that the model produced good average values (Table 2).

**Table 2:** Values of several descriptive statistics.

		Station st1			Station st2		
		obs	mod300	mod100	obs	mod300	mod100
	Mean (m/s)	0.2926	0.3422	0.3377	0.3263	0.3381	0.3023
	Minimum (m/s)	0.0072	0.1241	0.1154	0.0048	0.1367	0.078
Current Intensity (m/s)	Maximum (m/s)	0.6448	0.5413	0.5881	0.6027	0.5330	0.5279
	Variance (m/s)	0.0245	0.0117	0.0187	0.0117	0.0123	0.0198
	Sta dev (m/s)	0.1564	0.1121	0.1367	0.1084	0.1108	0.1408
	IoAd		0.75	0.67		0.83	0.64
	RMAE		0.16	0.15		0.04	0.07
							
Elevation (m)	IoAd	0.987					
	RMAE	0.03					

The IoAD and RMAE of water current intensity and elevation measured (obs) and modelled (mod) at the seabed at two stations (st1, st2).

**Figure 4:**
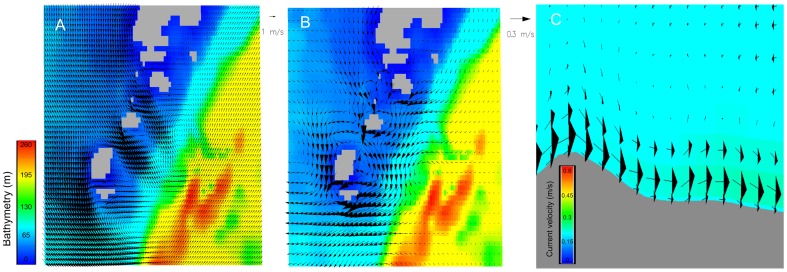
Horizontal water circulation in the Mingulay Reef Complex. (a) Snapshot of the seabed current from the model simulation (11/07/06). Arrows illustrate the direction and speed of the current. A very clear circulation structure can be indentified with three anticlockwise eddies in different areas in the islands. (b) The residual circulation in the seabed layer during the period modelled. (c) the downwelling at Mingulay Area 1 reproduced using this high resolution 3D hydrodynamic model (vertical exaggeration, 4x).

Our model reproduced the tidally-driven current regime and showed several eddies due to tidal current jets entering the area through the channels between the islands of Barra and Mingulay (Figure 4a). These jet currents influence the internal circulation structure. A circular current regime can be observed, with current direction flowing south then turning to the west and back again through the channels at the end of the Hebridean islands in the Mingulay area). A drifter could confirm this modeled circular structure around the Mingulay island (see www. http://martech.sams.ac.uk/fastnet/map_all.php, drifter number 25). In the east of the study area, the residual current calculated during a full tidal cycle is predominantly northward whereas in the westernmost portions, closer to the Hebridean island chain, the residual currents flow in a southward direction (Figure 4b). The hydrodynamic model successfully recreated the current regime, as well as downwelling phenomena [16] observed *in situ* at Mingulay Area 1 (Figure 4c). The model also provided evidence of downwellings in Mingulay 5 and Banana Reef with the same characteristics as describe in Mingulay 1 (Figure 4c). These several downwelling phenomena occurs with ∼30–60 minutes delays, but is difficult to ascertain the timings with high precision.

**Figure 5:**
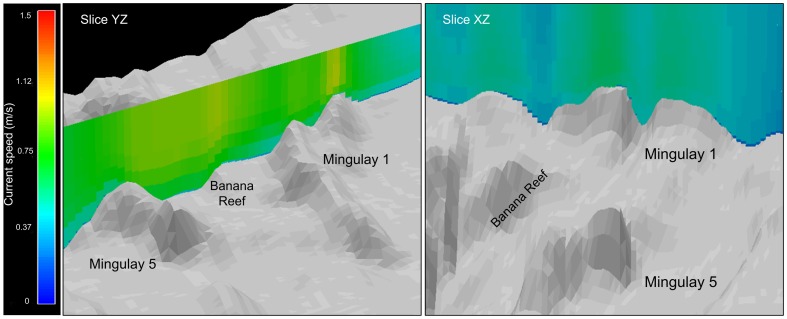
Vertical water circulation in the Mingulay Reef Complex. Vertical current velocity snapshots covering the whole Reef (Slice YZ) and in detail at Mingulay 1 (Slice XZ) with a 3x vertical exaggeration.

We can observe areas of high-speed current velocity in the whole water column in several areas in the Mingulay Reef Complex (Figure 5, slice YZ) creating high turbulence and enhancing the downwelling phenomena. Differences can also be noticed in the current speed at different water depths and lateral areas in Mingulay 1 (Figure 5,slice XZ).

**Figure 6:**
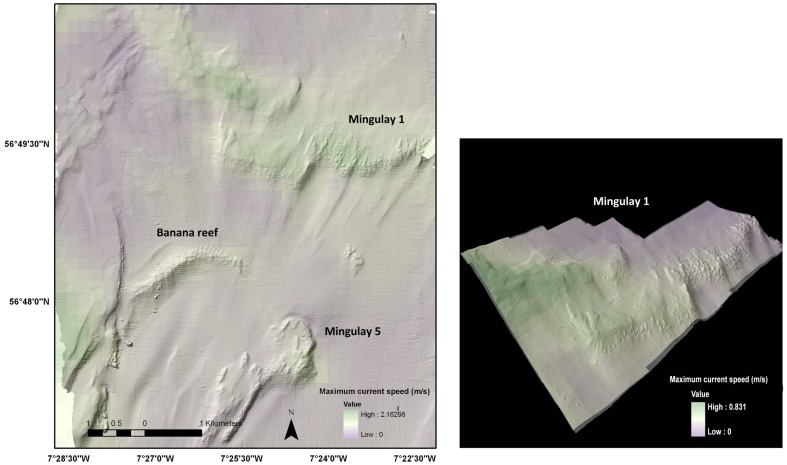
Example of integration between the hydrodynamic model in 3D GIS. The shading illustrates the maximum current velocity at the seabed across the Mingulay reef complex (plan view, left) and across Reef Area 1 (3-D view with vertical exaggeration (2x), right).

The mean, maximum and the standard deviation values of the modelled current velocities were incorporated into GIS to provide an adaptable graphical user interface for 2D, 3D and temporal visualization (see Figure 6). The results show a change in current velocity from west to east with higher current speeds in the western areas closer to the island chain. High current speed areas were found in Mingulay 1, Mingulay 5 and Banana Reef.

**Figure 7:**
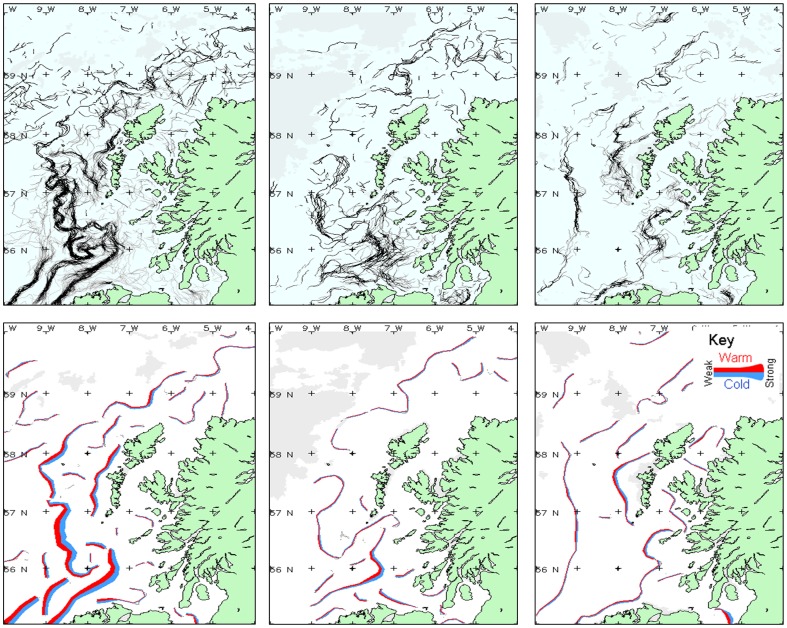
Remote Sensing I. Thermal fronts in several months in 2010. Thermal fronts observed during selected months in 2010. Left column: March; center column: May; right column: December. The upper maps show the composite front map derived from all cloud-free observations during that month; whereas the lower maps depicts a simplified version of the fronts, coloured red-blue to indicate the warm-cold side of the front, and the thickness of the line indicates the strength of the front.

Figure 7 shows thermal fronts observed over a wide region during selected months of 2010, in order to illustrate seasonal differences. In March the fronts had a strong thermal signature in the Irish Sea and on the Hebrides section of the continental shelf. In May, a weak thermal front was close to Mingulay Reef Complex, and in December several fronts were observed but were generally weak in different ocean areas.

**Figure 8:**
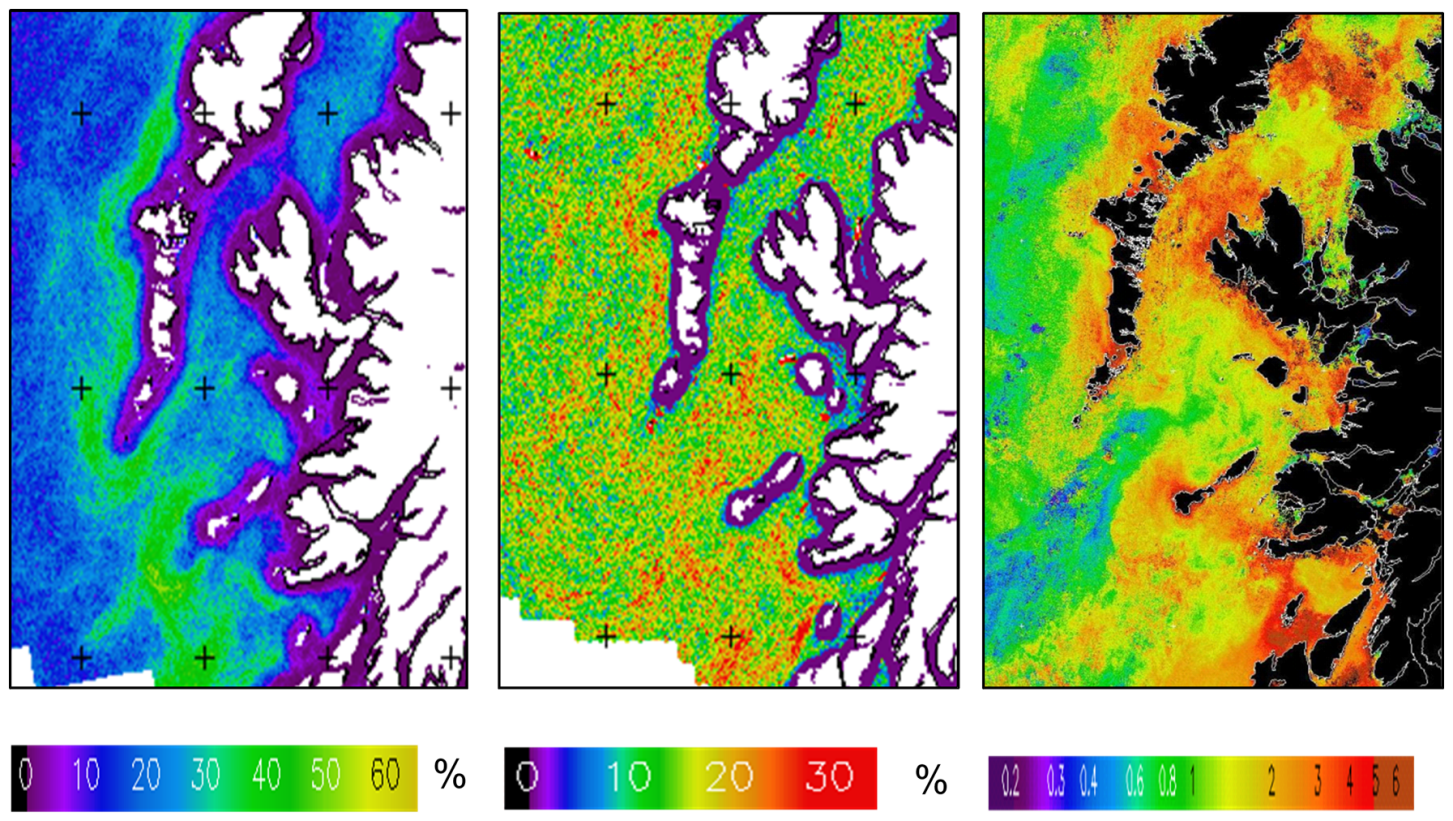
Remote Sensing II. Mean all-season thermal and Chl-*a* fronts. Mean all-seasons front frequency for the Outer Hebrides and Tiree: (a) thermal fronts; (b) Chl-a fronts. (c) MERIS Full-resolution (300 m) resolution chl-a composite for 23–30 Apr. 2011 showing chl-a fronts in similar location s to frequent thermal fronts.

The ocean fronts observed in this region by satellite were primarily thermal, concentrated in the areas west of the Hebridean islands and the isle of Tiree (Figure 8a). There was a slightly increased frequency in the Aqua-MODIS 1 km Chl-*a* fronts in areas affected by thermal fronts (Figure 8b).

## Discussion

### Ecohydrodynamics

Relevant temporal scales vary from less than a second, dominated by diffusion and turbulence, to months over which tidal regimes and major oceanographic phenomena come into play. Relevant spatial scales vary from centimeters where flow within and across corals affects local biogeochemical fluxes and skeletal morphologies up to kilometers across entire reef complexes.

Ecohydrodynamics are widely accepted as key drivers of marine ecosystem structure and functioning in deep water settings such as seamounts, ridges and canyons, but empirical data and models of complex flow are still largely lacking.

Over this broad region, the existence of fronts (Figure 7 and 8 a,b) had been verified by several studies [23],[24],[26],[54]–[56].A thermal front is usually present on the Scottish shelf during spring and summer, marking the boundary between the cooler tidally-mixed water near to the coast, and the warmer stratified surface water further offshore. In December there are also fronts visible along the shelf-break, probably related to the slope current. However, the distribution of chl-a fronts is not as clear. This could be explained by the position of chl-*a* fronts being more variable than the physical features, or a low surface chl-*a* concentration; there may be a deep chl-*a* maximum that cannot be detected using EO data. However, ocean colour fronts may be observed occasionally, as in this example from April 2011 (Figure 8c), where there was a sharp contrast between the nutrient-rich shelf water and less productive oceanic water during the spring bloom. The front southwest of Tiree is related to the shallow inner shelf that extends southwest from the island and causes tidal mixing [56].

We observed thermal fronts and peaks of chl *a* values that may influence the coral reef areas in the Hebrides Sea. The elevated chl-*a* distribution in surface coastal water found during spring suggests the phytoplankton bloom began near the Scottish mainland, Little Minch and the Sea of the Hebrides. These high productivity areas and the majority of the thermal fronts are outside or very close to the model boundaries, and for these reasons linking information between the remote sensing features and the model outputs are problematic. Our modelled time scale is ∼ one tidal cycle (15 days) whereas the oceanographic features could range from hours to months. Additionally, the intrusion of Atlantic water into the Sea of the Hebrides is not recreated by the model. However we can assume that the currents, the downwelling, and the residual current regime in a southward direction (as indicated by the model) suggests that the reef areas can be feed by phytoplankton and particles from the Little Minch and Hebrides coastal areas. Due to the proximity of the model boundary to Tiree Island, our model cannot provide specific information about the thermal fronts in this area.

Previously only two studies used hydrodynamics to model ecological dynamics on cold-water coral reefs [15],[57] with coarse and reasonable high spatial resolution. We have developed and applied the first high resolution (100 m) temporally dynamic 3D ocean model to improve our understanding of how regional ecohydrodynamics affect cold-water coral reef ecosystems. The hydrodynamics of our study area had been previous modeled [58],[59] with different spatial resolutions provided information about the influence of tide, wind and upstream inflow. The tidal residual flow modelled in our study matched the patterns and directions of previous study [59].

Although numerical modelling was challenging due to the scarcity of information for 3D model parameterization, several open boundary extensions were tested, and all available hydrographic data was used for validation (assuming barotropic flow is a good characteriser of hydrodynamic regime). Subsequent comparison between predicted and ground-truthed measurements of what is a hydrodynamically complicated area showed good agreement. However, it is important to remember the importance of taking density-driven dynamics into consideration. The next steps will be to advance the state-of-the-art by developing a fully three-dimensional baroclinic hydrodynamic model as suggested by Hill et al. [29] across a longer time period to better characterise the residual currents and extend the open boundaries for a better understanding of oceanographic features such as thermal and chlorophyll fronts.

Some studies [27],[29] provided evidence of an intrusion of Atlantic water into the Sea of the Hebrides and found that during summer each year surface heating over the isolated regions with weak tidal stirring such as the topographic depressions found in the area could lead to the formation of domes of cold, dense bottom water trapped beneath the thermocline [29]. Our model suggests that the Mingulay Reef Complex has a very strong tidal current regime and that water exchange is too rapid to actually account for such a cold lens.

The ecohydrodynamic temporal scale at Mingulay Reef complex range is about hours to minutes dominated by the tidal regime that affect the coral. The downwellings every 6 hours and a high current tidal regime are the key oceanographic features. The hydrodynamic model recreated a realistic tidal current regime and the downwelling phenomenas [16], which now provides us with the basis to model and predictively map areas with dense coral growth that also have high coral-associated biodiversity of sessile suspension feeders at the complex [34], [35].

Recently a study considered that a downwelling and mixing play an important role in the nutrition of cold-water corals on a fjordic sill in the Norwegian Skagerrak as well as reef geomorphology [60]. The role of downwelling of surface matter was also confirmed at Mingulay, where *Lophelia* feeds on herbivorous copepod crustaceans [18] and surface algal matter [37]. The current regime in the Sea of the Hebrides transports zooplankton from the surrounding areas to the coral reef mainly from the Little Minch, and advects particles from high productivity areas in the west coast of the Hebrides island chain. When the inshore-offshore distribution of plankton species on the west coast of Scotland were compared, it was found that a large oceanic copepod species was more abundant with increasing distance from the mainland [61]. In addition, current velocity affects food uptake by *Lophelia pertusa*, with several studies suggesting velocities values between 2.5 cm/s to 5 cm/s as an optimal range for prey capture, although the range could exceed 40 cm/s [37],[62],[63]. Our results suggest that the current regime with high velocities, created high turbidity that can play an important role advecting food particles to the corals, and that the area has an active sediment transport with strong local near-bed currents preventing the coral to be buried.

Drifting buoys drogued at 15 m depth revealed that seawater density plays an important role in the Scottish coastal circulation with a significant contribution to the northward transport and recirculation patterns [29]. These features were predicted to have great significance for larval transport including for commercially important species such as the Norway lobster (*Nephrops norvegicus*), and may also be relevant to the distribution of *Lophelia* off Mingulay. In the case of cold-water corals, a seawater density envelope of sigma-theta between 27.35 and 27.65 kg m^−3^ could act as a physical boundary condition for coral growth and distribution depending on larval buoyancy [64]. Little information exists about the timing of gamete release, larval competency and recruitment in deep water scleractinians [65], however reproductive particles could behave as passive tracers with water density playing an important role [29]. A fully baroclinic 3D hydrodynamic model coupled with a particle tracking model could help to explain larval transport and the distribution of these species.

### Ecosystem-scale relevance

In addition to close coupling between hydrodynamic regimes and the growth and distribution of cold-water corals, ecohydrodynamics shape the structure and function of biological communities inhabiting the reef complex. Species diversity and composition of these communities change in response to interactions between hydrography and seabed terrain [35]. Current speed was particularly important to community assembly, with few fauna being able to withstand an exposed high current speed environmental niche. These interactions are also important for local sharks, which seem to spawn in areas not directly exposed to strong currents but with adequate water motion to ventilate egg capsules deposited by pregnant females [36]. We believe that our hydrodynamic model has already provided valuable additional input to ecological models of marine biodiversity [35] and could be used in future studies with conservation applications, for example in models that predict coral larval dispersal trajectories and reproductive output.

### Conclusions and future perspectives

Once more satellite data has been collected under future research programmes, connectivity between satellite and modelled data can be improved. The results from the hydrodynamic model here have been incorporated into GIS to provide an adaptable graphical user interface for 2D, 3D and temporal visualization for interrogation of results and as an input to other spatial models or decision support systems. This integration also offers the possibility of combining the data with layers of spatial information about economic and social aspects, communications and security of the study area to develop an integrated socio-ecological approach for the selection of marine protected areas. The process of 3D circulation modelling is challenging; however the benefits of such a decision support tool or modelling future climate change scenarios are also considerable.

The oceans have entered a period of rapid change with rates of warming and acidification forecasted to reach unprecedented levels. In almost all marine ecosystems we lack a rigorous modelled framework to assess the ecological implications of predicted change. It is recognised that at present rates of carbon dioxide release the depth of the Atlantic's aragonite saturation horizon will shoal rapidly in the coming century [17],[66]. Recent work shows that the downwelling at the Mingulay Reef Complex brings more alkaline surface waters potentially helping mitigate the overall trend of declining saturation states [67]. Future models need to account for this natural variability and attempt to incorporate carbonate chemistry and other key parameters if we are to achieve a properly integrated picture of the ramifications of global ocean change. Similarly, ecohydrodynamic approaches will be important in fully understanding the implications of marine renewable energy and related offshore installations.
