# Correction: Ecohydrodynamics of Cold-Water Coral Reefs: A Case Study of the Mingulay Reef Complex (Western Scotland)

**DOI:** 10.1371/journal.pone.0106208

**Published:** 2014-08-19

**Authors:** The PLOS ONE Staff

The second author’s name is spelled incorrectly. The correct name is: Peter I. Miller. The correct citation is Navas JM, Miller PI, Henry L-A, Hennige SJ, Roberts JM (2014) Ecohydrodynamics of Cold-Water Coral Reefs: A Case Study of the Mingulay Reef Complex (Western Scotland). PLoS ONE 9(5): e98218. doi:10.1371/journal.pone.0098218
